# Depression, Anxiety, Stress, and Pain Severity in Patients With Recurrent Aphthous Stomatitis: A Cross-Sectional Study

**DOI:** 10.7759/cureus.62694

**Published:** 2024-06-19

**Authors:** Rohan Shinkre, Ishan Mukherji, Aarya Bharadwaj, Nikhil V Suresh, Ankita Dutta Banik, Sneha Jaiprakash Pednekar, Srivastava B K, Shruthi Eshwar, Parimala Rajagopal

**Affiliations:** 1 Central Research, KLE Society's Institute of Dental Sciences, Bengaluru, Bengaluru, IND; 2 Public Health Dentistry, Guru Nanak Institute of Dental Sciences and Research, Kolkata, IND; 3 Public Health Dentistry, KLE Society's Institute of Dental Sciences, Bengaluru, Bengaluru, IND; 4 Dentistry/Oral Medicine and Radiology, Karkinos Healthcare Private Limited/Medella-Karkinos Oncology Institute, Kolkata, IND

**Keywords:** anxiety, stress, recurrent aphthous stomatitis, pain, depression

## Abstract

Background

Recurrent aphthous stomatitis (RAS) is a chronic oral ulcerative condition with an elusive etiology that is associated with excruciating pain. Psychological factors have been suspected as a possible risk factor in its onset and development. Depression, anxiety, and stress play pivotal roles in how people experience pain. Hence, we aimed to explore the relationship between depression, anxiety, stress, and the severity of pain perceived due to recurrent ulcers in RAS patients.

Materials and methods

A cross-sectional comparative study was conducted on 248 patients, comprising 124 diagnosed with RAS and 124 healthy subjects without this oral condition. Patients from both of these groups were matched with regard to age and sex and recruited over a period of one year from a dental hospital in Bengaluru, India. Patients with any other oral lesions, painful oral conditions, or systemic and psychological illnesses were excluded. Depression, anxiety, and stress were assessed in these patients using the Depression Anxiety Stress Scale 21 (DASS-21). Utilizing a Visual Analog Scale, ulcer-related pain was assessed in patients with RAS. The data were analyzed using the chi-square test and Pearson’s correlation test in IBM SPSS Statistics for Windows, Version 26.0 (released 2019, IBM Corp., Armonk, NY).

Results

The chi-square analysis showed that participants with RAS showed a statistically significant higher prevalence of extreme stress (n = 39, 31.5%), extreme anxiety (n = 82, 66.1%), and depression (n = 38, 30.6%) as opposed to those without this oral condition. A moderately positive correlation was observed in the Pearson's test between the severity of pain experienced and stress and anxiety (r = 0.65 and 0.60, respectively, p < 0.05), while a mild positive correlation was observed between the severity of pain and depression (r = 0.35, p < 0.05).

Conclusion

Depression, anxiety, and stress influenced the severity of pain in these lesions, dictating the need for a holistic approach that integrates psychological interventions in the management of such chronic oral conditions with psychological links.

## Introduction

Mental health is a cornerstone of human thriving, influencing how we think, feel, communicate, and live fulfilling lives [1]. Earlier, the mind and body were viewed as separate beings. However, research in the past decades has swayed the opinion to the contrary. Several studies summarized in a review by Biondi et al. [2] reported that psychological distress caused well-defined biochemical and neuro-endocrine changes. It sheds light on the effect of psychological factors on the immune system and their significance in the initiation and progression of immunological illnesses manifesting in various parts of the body [2].

In many systemic disorders, physical or psychological dysfunction can manifest in the oral mucosa [3]. Recurrent aphthous stomatitis (RAS) is one such chronic immune-mediated oral pathology that is widely discussed in this regard. The primary clinical manifestations of this illness are recurrent solitary or multiple ulcers limited to the non-keratinized oral mucosa, with inflamed, elevated edges and a necrotic center. These ulcers are associated with intense pain. Various factors, such as positive family history, local trauma, hypersensitivities, dietary inadequacy, immunological disruption, and smoking, have been linked to the pathophysiology of RAS. Psychological factors, such as anxiety, stress, and depression, have gained traction in this regard as they hold significance in both the onset and continuation of RAS ulcers [4-7].

Depression, anxiety, and stress all play imperative roles in how people experience pain. Chronic pain and mood disorders, such as depression, anxiety, and stress, have a bidirectional relationship in which each influences the other [8-10]. Hence, comprehending the complex interplay between pain, depression, anxiety, and stress is pivotal for comprehensive treatment strategies for individuals with chronic pain.

As RAS is a chronic, painful illness, comprehending the association between psychological factors related to its elusive etiology and ulcer-related pain severity is crucial for designing focused therapeutic strategies and improving patient outcomes. Hence, the purpose of this study was to investigate whether the prevalence of stress, anxiety, and depression affect the level of pain that RAS patients experience due to these recurring oral ulcers.

## Materials and methods

Study setting and population

This cross-sectional comparative study included adults over 18 years of age visiting the outpatient department of KLE Society’s Institute of Dental Sciences, Bengaluru, India, using the convenience sampling method from October 1, 2020, to November 30, 2021. Participants were recruited into two groups. Patients diagnosed with RAS ulcers constituted the study group. The comparison group was comprised of patients without RAS ulcers that were matched to the patients in the RAS group with regard to age and sex. Individuals who were uncooperative, had systemic disorders or mental illnesses, were on psychoactive medicines, were receiving treatment for RAS, or had any other oral lesions or painful oral conditions were excluded. Prior ethical clearance was obtained from the KLE Institutional Ethics Committee (KIDS/IEC/NOV-19/33), and the study was carried out as per STROBE (Strengthening the Reporting of Observational Studies) guidelines (see Appendix).

Sample size estimation

A sample size of 124 for the two groups was computed using the formula N = [(Zα+Zβ)/C]2 + 3, where Zα​ = 1.96, Zβ​ = 0.84, and C was taken as 0.25.

Data collection

Demographic variables of age and sex and the medical history of the patients were recorded before the clinical examinations. An experienced oral medicine specialist used sterile mouth mirrors to diagnose recurrent aphthous ulcers based on the clinical appearance, symptoms, and history of recurrence of these ulcers.

The Depression Anxiety Stress Scale-21 (DASS-21) [11] was handed out to the patients from both groups in person to evaluate their levels of depression, anxiety, and stress. The patients were requested to fill out the forms themselves. The self-reported responses of the patients were noted. Patients with depression, anxiety, and stress were categorized as having mild to severe levels of these conditions based on the cumulative scores of the scale's items [11].

The Visual Analog Scale [12] was given in person only to patients with RAS to measure the pain level experienced on account of the lesions. Scores were self-reported by patients by marking them on a 10-cm pain scale ranging from "no pain" to "worst pain."

Statistical analysis

Descriptive statistics like mean and standard deviation, frequencies, and percentages were calculated for continuous and categorical variables, respectively. The normality of the data was determined utilizing Kolmogorov-Smirnov test. A chi-square test was adopted to compare the levels of depression, anxiety, and stress in the patients from the two groups. Pearson’s correlation test was used to check for any correlation between the Visual Analog Scale scores for the pain experienced by the RAS patients and the scores for depression, anxiety, and stress. Statistical analyses were performed using IBM SPSS Statistics for Windows, Version 21.0 (released 2019, IBM Corp., Armonk, NY) at a 5% level of significance.

## Results

The mean age of the 248 patients recruited in the study was 28.89 ± 9.25 years. In the RAS group, most of the patients were between the ages of 21 and 30, with the least in the 51-year-old and over-age groups. In the comparison group, a similar high prevalence of patients in the 21-30-year age group was found, and the least was seen in the 41-50-year age group. In the RAS group, more than 60% of the patients were females, while over 75% of the patients in the comparison group recruited were females (Table 1).

**Table 1:** Frequency distribution of the demographic variables among the patients with and without recurrent aphthous stomatitis (RAS)

Demographic variables		Recurrent aphthous stomatitis (RAS)	
		Present	Absent
Age groups	18-20 years	10 (8.1 %)	1 (0.8%)
	21-30 years	48 (38.7%)	95 (76.6%)
	31-40 years	32 (25.8%)	23 (18.5%)
	41-50 years	26 (21.0%)	2 (1.6%)
	51 years and above	8 (6.5%)	3 (2.4%)
Sex	Male	49 (39.5%)	27 (21.8%)
	Female	75 (60.5%)	97 (78.2%)

The data presented in the chi-square table demonstrates the distribution of stress, anxiety, and depression levels between the groups of patients with and without RAS. The chi-square statistic (67.56) with a p-value <0.05 showed a significant association between the presence of RAS and stress levels. Patients with RAS were more likely to experience stress, with a significant rise in the severe and extremely severe stress categories. The chi-square statistic (69.82) with a p-value <0.05 indicated a significant relationship between RAS and anxiety levels. Similar to stress, elevated anxiety levels, particularly in the extremely severe group, were exceedingly prevalent in those with RAS. The coefficient of chi-square (24.59) with a p-value <0.05 showed that patients with RAS had a greater prevalence of severe and extremely severe depression. Significant associations between RAS and elevated levels of stress, anxiety, and depression showed that the severity of these psychological variables is higher in patients afflicted with RAS (Table 2).

**Table 2:** Distribution of the levels of stress, anxiety, and depression between the patients with and without recurrent aphthous stomatitis (RAS)

Psychological Variables		Recurrent aphthous stomatitis (RAS)		χ2 statistic	p-value
		Present	Absent		
Stress	Absent	38 (30.6%)	92(74.2%)	67.56	<0.001
	Mild	5 (4.0%)	12 (9.7%)		
	Moderate	14 (11.3%)	8 (6.5%)		
	Severe	28 (22.6%)	9 (7.3%)		
	Extremely severe	39 (31.5%)	3 (2.4%)		
Anxiety	Absent	30 (24.2%)	77 (62.1%)	69.82	<0.001
	Mild	1 (0.8%)	5 (4.0%)		
	Moderate	6 (4.8%)	20 (16.1%)		
	Severe	5 (4.0%)	2 (1.6%)		
	Extremely severe	82 (66.1%)	20 (16.1%)		
Depression	Absent	59 (47.6%)	76 (61.3%)	24.59	<0.001
	Mild	7 (5.6%)	15 (12.1%)		
	Moderate	8 (6.5%)	16 (12.9%)		
	Severe	12 (9.7%)	6 (4.8%)		
	Extremely severe	38 (30.6%)	11 (8.9%)		

**Figure 1:**
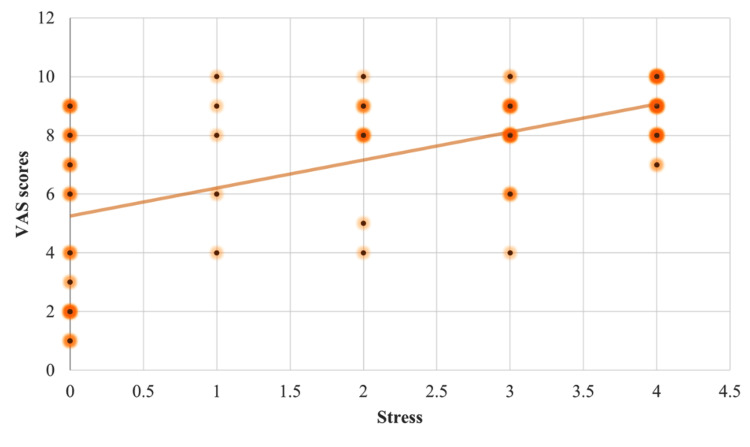
Scatter plot diagram depicting the correlation between the Visual Analog Scale (VAS) scores with the stress scores.

**Figure 2:**
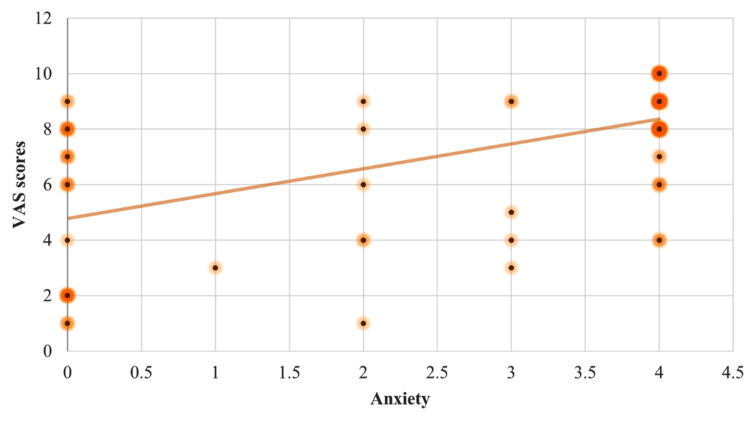
Scatter plot diagram depicting the correlation between the Visual Analog Scale (VAS) scores with the anxiety scores.

**Figure 3:**
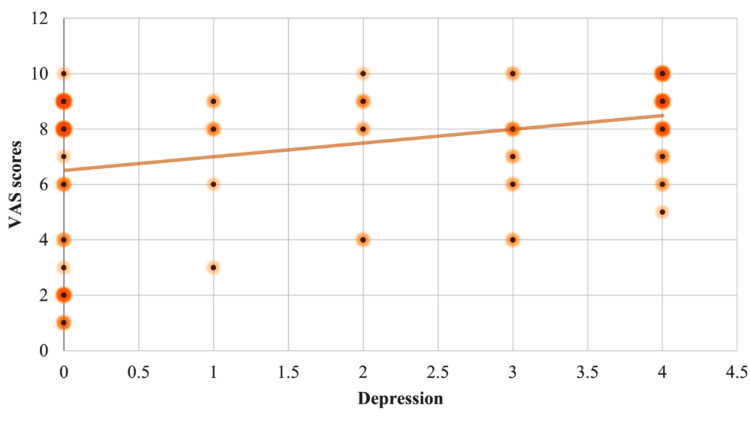
Scatter plot diagram depicting the correlation between the Visual Analog Scale (VAS) scores with the depression scores.

Individuals with aphthous ulcers reported a mean pain score of 7.35 ± 2.52 on the Visual Analog Scale. The Pearson's correlation test revealed a statistically significant positive association between stress and the severity of pain experienced in RAS, r (122) = 0.62, p = 0.01. Anxiety and severity of pain experienced due to the lesion also had a statistically significant strong positive connection, r (122) = 0.60, p = 0.01. It also revealed a positive link between depression and the severity of pain caused by the lesion, r (122) = 0.35, p = 0.01 (Figures 1-3).

## Discussion

The purpose of this study was to investigate how the prevalence of stress, anxiety, and depression affected the level of pain perceived in RAS, a chronic oral condition that has an elusive etiology and cannot be completely cured.

There was a higher prevalence of RAS in the age group of 21-30 years. These findings coincide with the results of a study by Akmal et al., in a south Indian population [13]. According to some studies, the majority of people with RAS had the ailment before the age of 30, with the condition peaking in their 20s. As one ages, the frequency of active lesions has shown a decline [4]. Females had a higher prevalence of the lesion in our sample than males. Previous studies also found that females were more likely than males to suffer from RAS, in both children and adults. Some studies attribute this greater prevalence of RAS in females to hormonal differences between the two sexes [4].

The results of our study revealed significantly escalated levels of depression, anxiety, and stress in the patients with RAS as opposed to those without the condition. These results were consistent with the findings of other studies [7,14], where elevated levels of depression, anxiety, and stress were observed in participants with a history of RAS. According to Gallo et al., psychological stress may serve as a trigger or moderating element in RAS-afflicted patients [15]. Another observational study showed RAS patients with elevated salivary and blood cortisol levels, as well as increased anxiety [16]. Stress and anxiety can boost salivary cortisol levels by activating the HPA (hypothalamic-pituitary-adrenal axis) and impact immune function, leading to inflammation through increased leukocyte activity, which may lead to episodes of recurrent aphthous ulcers in the oral cavity [4]. Ziaudeen et al. found that psychological stress in RAS patients disturbed the oxidant-antioxidant balance, leading to oxidative stress and the onset and persistence of aphthous ulcers [17]. Psychological factors like anxiety and depression have also been shown to alter immune reactions like stress, potentially contributing to the onset and persistence of RAS [14].

This study found a positive correlation between depression, stress, and anxiety levels and the intensity of pain among RAS patients. This was similar to the findings of a study conducted in a European cohort [18]. Depression, anxiety, and stress have a bidirectional relationship with chronic pain and mediate largely how people perceive and adapt to the pain. Mood disorders, particularly depression and anxiety, play a significant role in exacerbating pain perception in all clinical situations, as well as adversely affecting pain experience. There are physiological parallels between depression and chronic pain. For instance, the physical "descending inhibition" of pain perception is correlated with noradrenaline and serotonin, which are also involved in the pathophysiology of depression. These two neurotransmitters modify incoming pain impulses by acting in the periaqueductal regions and limbic systems. Depression and anxiety have been shown to mediate pain by heightening pain perception and decreasing pain tolerance [8-10,19]. By fusing nociceptive and stress-affect-related data in the central amygdala, chronic stress has been shown to intensify the pain [20].

Depression, anxiety, and stress are all complicated disorders with multiple causes. They can be caused by a variety of causes, including genetics, substance abuse, chronic stress, and triggers in the environment. Genetic variations have a substantial effect on predisposing people to illnesses, such as depression and substance abuse [21]. Substance abuse and stress-induced cognitive dysfunction have been shown to have a favorable and statistically significant impact on anxiety and depression [22]. Chronic or elevated stress, particularly in early life, is also predictive of depressive disorders through dysregulated HPA axis activity and inherited risk factors, emphasizing the intricate interplay between environmental stressors, familial histories, and epigenetic alterations in the development of depressive conditions [23]. Humans can experience stress from a variety of sources, including environmental, physiological, neurological, and psychological variables. Environmental stressors, such as abrupt changes, uncertainties, and dangers, can cause stress responses. Physiologically, elevated levels of stress have been detected associated with shorter telomeres, higher inflammatory markers, and modified cortisol responses [24].

Comprehending the connection between the mind and the body is the first step toward devising techniques that reduce the prevalence of co-occurring issues and provide aid to those struggling with mental illnesses, chronic conditions, or both. The findings of this study establish precedence for this as we found escalated levels of depression, anxiety, and stress in patients with RAS lesions. These psychological variables were positively correlated with the severity of the ulcer-related pain perceived in the RAS patients. Hence, the treatment of these underlying psychological disturbances may lead to improved clinical features in RAS patients. Conventional therapies that focus primarily on the conservative management of pain in RAS patients need to be revised to include psychological factors that dictate the clinical symptoms of these lesions. This would lead to a more effective and holistic management of this chronic painful oral condition. Future studies should look at comprehensively exploring the psychological underpinnings of RAS and the development of tailored psychotherapy interventions that are designed specifically to meet the unique needs of patients with this disease.

The external validity of our study results is attributed to its representative sample. However, there were certain limitations to our study. The study's cross-sectional design makes it challenging to ascertain the temporal associations between the three psychological variables and pain severity associated with this painful oral condition. Although the DASS-21 test is widely utilized in medical studies and practice for the assessment of the three psychological variables, the responses to these tests are self-reported. As a result, the patients completing them might easily inflate or decrease the final score leading to a response bias. Efforts were made to preclude this by ensuring the patients that their responses would be kept confidential.

## Conclusions

The study reveals that depression, anxiety, and stress influence the perception of ulcer-related pain in individuals with RAS. This emphasizes the necessity of integrating psychological aspects of patients into therapeutic and preventive treatments for RAS, in addition to pain management. Addressing the psychological components could minimize the frequency and severity of outbreaks, boost therapy adherence, and improve overall patient well-being. Future research should look into the psychological roots of RAS in addition to developing pertinent psychotherapy interventions to meet the distinct requirements of these patients.

## Appendices

**Table 3:** STROBE (Strengthening the Reporting of Observational Studies) statement: checklist of items that should be included in reports of cross-sectional studies *Give information separately for exposed and unexposed groups.

	Item No.	Recommendation	Page No
Title and abstract	1	(a) Indicate the study’s design with a commonly used term in the title or the abstract	1
		(b) Provide in the abstract an informative and balanced summary of what was done and what was found	1
Introduction			
Background/rationale	2	Explain the scientific background and rationale for the investigation being reported	2
Objectives	3	State specific objectives, including any prespecified hypotheses	2
Methods			
Study design	4	Present key elements of study design early in the paper	2
Setting	5	Describe the setting, locations, and relevant dates, including periods of recruitment, exposure, follow-up, and data collection	2
Participants	6	(a) Give the eligibility criteria, and the sources and methods of selection of participants	2
Variables	7	Clearly define all outcomes, exposures, predictors, potential confounders, and effect modifiers. Give diagnostic criteria, if applicable	1, 2
Data sources/ measurement	8*	For each variable of interest, give sources of data and details of methods of assessment (measurement). Describe comparability of assessment methods if there is more than one group	2
Bias	9	Describe any efforts to address potential sources of bias	7
Study size	10	Explain how the study size was arrived at	2
Quantitative variables	11	Explain how quantitative variables were handled in the analyses. If applicable, describe which groupings were chosen and why	2
Statistical methods	12	(a) Describe all statistical methods, including those used to control for confounding	2
		(b) Describe any methods used to examine subgroups and interactions	2
		(c) Explain how missing data were addressed	Not applicable
		(d) If applicable, describe analytical methods taking account of sampling strategy	Not applicable
		(e) Describe any sensitivity analyses	Not applicable
Results			
Participants	13*	(a) Report numbers of individuals at each stage of study—eg numbers potentially eligible, examined for eligibility, confirmed eligible, included in the study, completing follow-up, and analyzed	3
		(b) Give reasons for non-participation at each stage	Not applicable
		(c) Consider the use of a flow diagram	Not applicable
Descriptive data	14*	(a) Give characteristics of study participants (eg demographic, clinical, social) and information on exposures and potential confounders	3
		(b) Indicate the number of participants with missing data for each variable of interest	Not applicable
Outcome data	15*	Report numbers of outcome events or summary measures	4,5 and 6
Main results	16	(a) Give unadjusted estimates and, if applicable, confounder-adjusted estimates and their precision (eg, 95% confidence interval). Make clear which confounders were adjusted for and why they were included	Not applicable
		(b) Report category boundaries when continuous variables were categorized	3
		(c) If relevant, consider translating estimates of relative risk into absolute risk for a meaningful time period	Not applicable
Other analyses	17	Report other analyses done—e.g., analyses of subgroups and interactions, and sensitivity analyses	Not applicable
Discussion			
Key results	18	Summarise key results with reference to study objectives	6
Limitations	19	Discuss limitations of the study, taking into account sources of potential bias or imprecision. Discuss both direction and magnitude of any potential bias	7
Interpretation	20	Give a cautious overall interpretation of results considering objectives, limitations, multiplicity of analyses, results from similar studies, and other relevant evidence	6, 7
Generalisability	21	Discuss the generalisability (external validity) of the study results	7
Other information			
Funding	22	Give the source of funding and the role of the funders for the present study and, if applicable, for the original study on which the present article is based	7
